# Experimental Susceptibility of North American Raccoons (*Procyon lotor*) and Striped Skunks (*Mephitis mephitis*) to SARS-CoV-2

**DOI:** 10.3389/fvets.2021.715307

**Published:** 2022-01-12

**Authors:** Raquel Francisco, Sonia M. Hernandez, Daniel G. Mead, Kayla G. Adcock, Sydney C. Burke, Nicole M. Nemeth, Michael J. Yabsley

**Affiliations:** ^1^Warnell School of Forestry and Natural Resources, University of Georgia, Athens, GA, United States; ^2^Southeastern Cooperative Wildlife Disease Study, Department of Population Health, College of Veterinary Medicine, University of Georgia, Athens, GA, United States; ^3^Department of Pathology, College of Veterinary Medicine, University of Georgia, Athens, GA, United States

**Keywords:** SARS-CoV-2, COVID-19, raccoons, skunks, Mephitidae, zoonoses, wildlife, One Health

## Abstract

Recent spillback events of SARS-CoV-2 from humans to animals has raised concerns about it becoming endemic in wildlife. A sylvatic cycle of SARS-CoV-2 could present multiple opportunities for repeated spillback into human populations and other susceptible wildlife. Based on their taxonomy and natural history, two native North American wildlife species —the striped skunk (*Mephitis mephitis*) and the raccoon (*Procyon lotor)* —represent a high likelihood of susceptibility and ecological opportunity of becoming infected with SARS-CoV-2. Eight skunks and raccoons were each intranasally inoculated with one of two doses of the virus (10^3^ PFU and 10^5^ PFU) and housed in pairs. To evaluate direct transmission, a naïve animal was added to each inoculated pair 48 h post-inoculation. Four control animals of each species were handled like the experimental groups. At predetermined intervals, we collected nasal and rectal swabs to quantify virus shed via virus isolation and detect viral RNA via rRT-PCR and blood for serum neutralization. Lastly, animals were euthanized at staggered intervals to describe disease progression through histopathology and immunohistochemistry. No animals developed clinical disease. All intranasally inoculated animals seroconverted, suggesting both species are susceptible to SARS-CoV-2 infection. The highest titers in skunks and raccoons were 1:128 and 1:64, respectively. Low quantities of virus were isolated from 2/8 inoculated skunks for up to day 5 post-inoculation, however no virus was isolated from inoculated raccoons or direct contacts of either species. Neither species had gross lesions, but recovering mild chronic pneumonia consistent with viral insult was recorded histologically in 5/8 inoculated skunks. Unlike another SARS-CoV-2 infection trial in these species, we detected neutralizing antibodies in inoculated raccoons; thus, future wildlife serologic surveillance results must be interpreted with caution. Due to the inability to isolate virus from raccoons, the lack of evidence of direct transmission between both species, and low amount of virus shed by skunks, it seems unlikely for SARS-CoV-2 to become established in raccoon and skunk populations and for virus to spillback into humans. Continued outbreaks in non-domestic species, wild and captive, highlight that additional research on the susceptibility of SARS-CoV-2 in wildlife, especially musteloidea, and of conservation concern, is needed.

## Introduction

As severe acute respiratory syndrome coronavirus 2 (SARS-CoV-2) continues to circulate on a global scale, the need to identify potential animal reservoirs, especially among wildlife, has become a priority, spurring surveillance and susceptibility trials of numerous species (1–15). Indeed, the COVID-19 pandemic has highlighted the need for a global One Health approach to address its solution (16). Wildlife health assessments, pathogen surveillance, and experimental trials are intrinsic components of this approach which have been neglected until recent decades, but have been integral in understanding the epidemiology of other recent pandemics such as severe acute respiratory syndrome (SARS) and middle eastern respiratory syndrome (MERS) (17–19).

Ongoing spillover events from humans to pets (e.g., dogs and cats), commercial animals (e.g., mink), and captive wildlife (e.g., tigers, gorillas) have raised concerns about the ability of SARS-CoV-2 to become endemic in abundant native wildlife species (20–22). A sylvatic cycle of SARS-CoV-2 could present multiple opportunities for repeated spillback into human populations and susceptible wildlife species. While the role of free-living wildlife in the emergence of SARS-CoV-2 remains unclear, the susceptibility and potential of a wildlife species as a reservoir could hold substantiable implications, not just for public health, but for the management, research, rehabilitation, and conservation of other susceptible animal species (23). In North America, several members of the Musteloidea (Mustelidae, Mephitidae, and Procyonidae) families are both taxonomically and ecologically relevant and likely have a high probability of becoming exposed to, infected with, and developing clinical disease to SARS-CoV-2 (24–28). In fact, ferrets (*Mustela putorius furo*), close relatives to North American Musteloidea, are well-established animal models for SARS (29–32) and are highly susceptible to SARS-CoV-2 (8–10, 33–38). Another close relative, mink (*Neovison vison*), is also highly susceptible to SARS-CoV-2 in experimental inoculations trials, as well as natural infections in commercial farms (5, 39–43). Most recently, Asian small-clawed otters (*Aonyx cinereus*) in a zoological institution were infected with SARS-CoV-2 (44). Like ferrets, both mink and otters experienced varying levels of respiratory disease upon infection with SARS-CoV-2. Unlike ferrets, both species were first found to be susceptible after transmission from an infected human caretaker, highlighting the anthropozoonotic potential of this virus (45).

**Figure 1:**
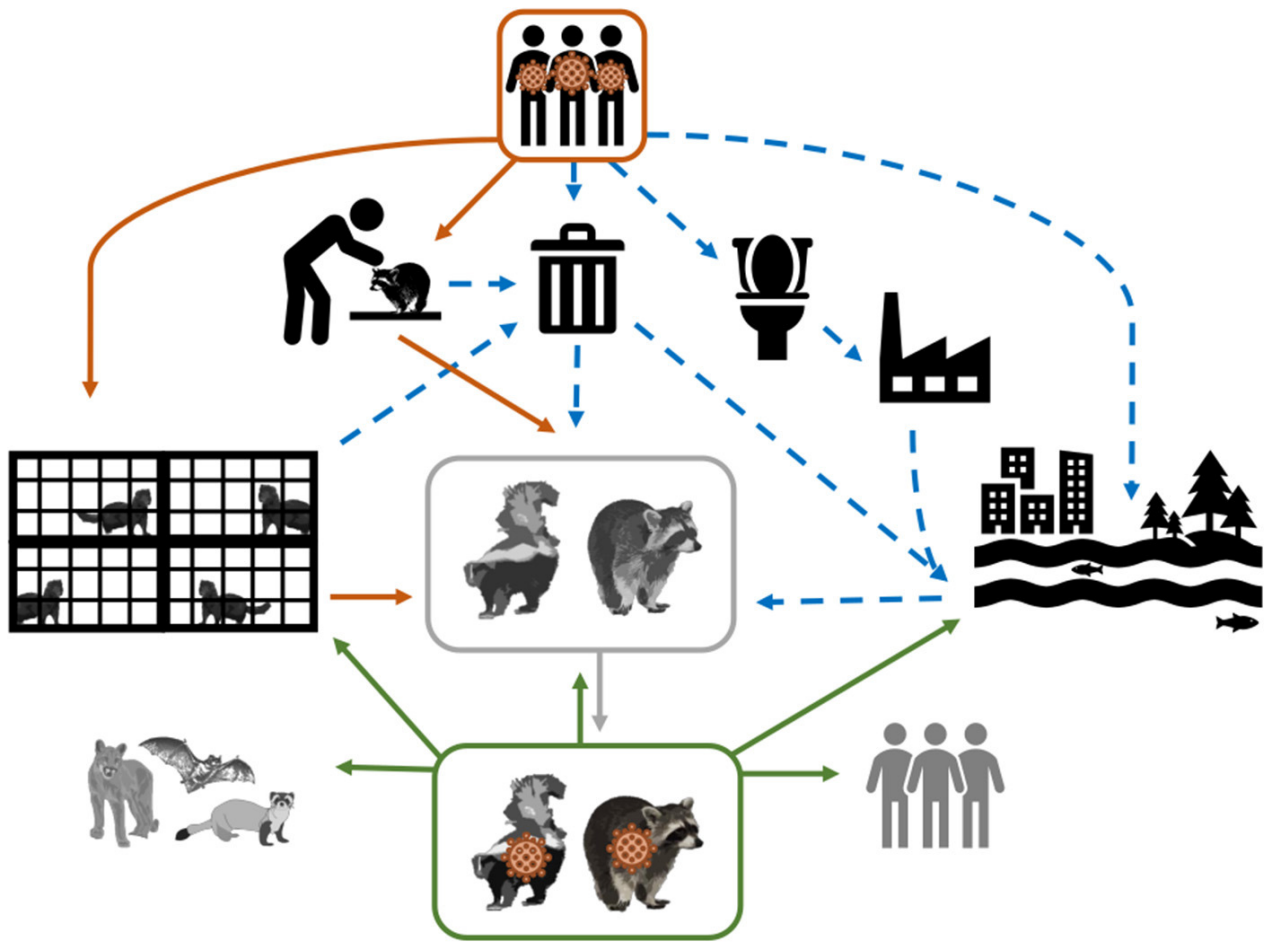
Conceptual model of the mechanisms of SARS-CoV-2 transmission from infected humans (direct transmission = solid orange arrows; and indirect transmission = dashed blue arrows) to susceptible wildlife (represented in greyscale). As depicted, SARS-CoV-2 shed by humans can be directly transmitted through activities that require handling and close contact (e.g., research and wildlife rehabilitation), or commercial operations (e.g., fur farms); however virus shed by humans could make its way into the environment via garbage (i.e., medical waste and household waste) and sewage. The solid gray arrow represents the establishment of SARS-CoV-2 in a wildlife species. The hypothesized spillback from this SARS-CoV-2 wildlife reservoir to susceptible human populations and other wildlife species is demonstrated by the solid green arrows.

Two Musteloidea, striped skunks (*Mephitis mephitis*, Mephitidae) and raccoons (*Procyon lotor*, Procyonidae), range throughout much of North America, and are abundant, opportunistic, omnivorous generalists (46). Raccoons are also well established in regions of Europe and Asia (47). Both species have become habituated to seek food and shelter near human homes, resulting in frequent interactions with domestic animals, humans, and their waste. Figure 1 depicts several hypothetical pathways from which SAR-CoV-2 can be transmitted from humans, both directly and indirectly, to species that have ample ecological opportunity, such as raccoons and skunks, justifying their importance as potential reservoirs. Skunks and raccoons are already notorious reservoirs of viruses that have substantial impacts on other wildlife and humans (i.e., rabies virus, canine distemper virus, protoparvoviruses) (48, 49). To determine their role in the epidemiology of SARS-CoV-2, this study evaluated the susceptibility to infection, seroconversion, transmission potential between conspecifics, tissue tropism, and pathology associated with SARS-CoV-2 in striped skunks and raccoons.

## Methods

### Animals and Husbandry

Sixteen juvenile (~10 week old), equal numbers of both sexes, captive-bred raccoons and skunks were obtained from a commercial, captive breeding animal facility in June and July 2020, respectively. Animals were either housed at the University of Georgia either in a Biosafety Level 2 (BSL-2) facility (control skunks) or in the Animal Health Research Center (AHRC; experimentally inoculated animals of both species and control raccoons) which is a high-security biocontainment facility. All of the experimental infection work was conducted under Biosafety Level 3 (BSL-3) protocols. All procedures involving the handling of animals and the SARS-CoV-2 virus were reviewed and approved by the University of Georgia's IACUC committee (A2020 04-016) and Office of Biosafety (2020 0048).

Animals were housed at ~21°C and 50% humidity. Both species were fed daily with commercially available omnivore diet (Mazuri® Omnivore Diet, Purina Mills, LLC., USA) and offered water *ad libitum*. The diet was supplemented by various fresh greens and protein items such as boiled eggs. Animals were identified by purposely shaved patches of fur either on the left, right, or center of their rump. Prior to inoculations, nasal swabs, rectal swabs, and blood samples were collected and tested by microtitration serum neutralization (SN) and virus isolation (VI) to ensure animals were not currently or previously infected with SARS-CoV-2.

### Experimental Design

Experimental animals (*n* = 12), excluding the control animals (*n* = 4) who were housed separately, were separated into 2 identical dosing groups with equal sexes per group. Each dose group consisted of four animals housed in pairs in two adjacent stainless-steel wire mesh cages (~1.5 x 1.5 x 2m). The high (H) and low (L) dose animals were intranasally inoculated with 10^3^ PFU and 10^5^ PFU of SARS-CoV-2 (*n* = 4 per dose, per species), respectively. The 10^5^ PFU dose has produced infections in ferrets and other species (9, 10). The 10^3^ PFU dose was used to mimic the amount of virus to which these species may be naturally exposed (e.g., through consuming human garbage or potentially animal-to-animal) and has also resulted in infections and clinical disease in ferrets (31, 37). Each animal was identified by a unique combination of numbers and letters that corresponded with their dosage group, their enclosure number, and the side where a section of their fur was shaved (i.e., raccoon H1L equated to high-dose raccoon from group 1 that shaved on the left side). All four experimental dose groups were housed in the same BSL-3 Agriculture (BSL-3Ag) room but were separated by approximately 6 meters and the directional air flow in the room flowed from the low to the high dose group (Supplementary Figure 1). The design of the BSL-3Ag facility does not allow for recirculated air, facilitating 13 to 15 air changes per hour, thus the likelihood of aerosol transmission between each group is negligible. To test for direct contact transmission, a single naïve conspecific was introduced to each pair of directly inoculated animals 48 h after inoculation. Control animals (*n* = 4) were housed in either a separate BSL-3Ag room (raccoons) or BSL-2 facility (skunks).

### Virus and Inoculations

The SARS-CoV-2 isolate used was USA-WA1/2020 which was originally isolated from a middle-aged male in Washington, USA who traveled to Wuhan China in January 2020. Skunks and raccoons were inoculated with 5^th^ passage virus. The virus was grown in vero-E6 cells (American Type Cell Culture [ATCC] Cat# CRL-1586, RRID:CVCL_0574) which were maintained in minimal essential medium (MEM, 5 L deionized water, 48 g of Minimal Essential Media Eagle (Sigma-Aldrich, Co., USA), 11.11 g bicarbonate) supplemented with 50 mL/L of iron fortified calf serum (Sigma-Aldrich, Co.) and 20 mL/L of Antibiotic Antimycotic Solution (10,000 units penicillin, 10 mg streptomycin, 25 μg amphotericin per mL). All cultures and microtitrations were incubated in a 5% CO_2_ atmosphere and 37°C.

For procedures, such as inoculation and venipuncture, raccoons and skunks were anesthetized with a combination of dexmedetomidine (0.04 mg/kg) (Dexdomitor™, Orion Corporation, Finland) and butorphanol (0.2 mg/kg) (Torbugesic™, Zoetis Manufacturing and Research, Spain), intramuscularly (IM), and reversed with atipamezole (0.25 mg/kg) (Revertindine™, Modern Veterinary Therapeutics, Germany) and naloxone (0.02 mg/kg) (Wintac Limited, India) given IM to return them to pre-anesthetic function as rapidly as possible.

The intranasal inoculations were performed on anesthetized animals using a 21-gauge catheter attached to a 1 mL luer slip syringe (BD Syringe, Becton, Dickinson and Company, USA). Experimental animals that were intranasally inoculated with live virus (*n* = 8) will be referred to as the directly inoculated (DI) animals or groups. A single direct contact (DC) animal was introduced to each pair of DI animals 48 h post-inoculation to evaluate direct transmission.

### Sampling

Animal health status (i.e., mentation, attitude, physical appearance, consumption of food) was evaluated twice daily. All animals were weighed at admission, and additional body weights were recorded for all animals on the days when they were fully anesthetized for venipuncture. Rectal temperatures were collected from all animals when anesthetized for venipuncture, normothermic was considered 37.2 to 39.2°C (99.0 to 102.5°F) for both species (50, 51). To collect serum, 2 mL of blood was drawn from the jugular vein and added to plain sterile vacutainer tubes (3 mL; Covidien™, USA).

**Figure 2:**
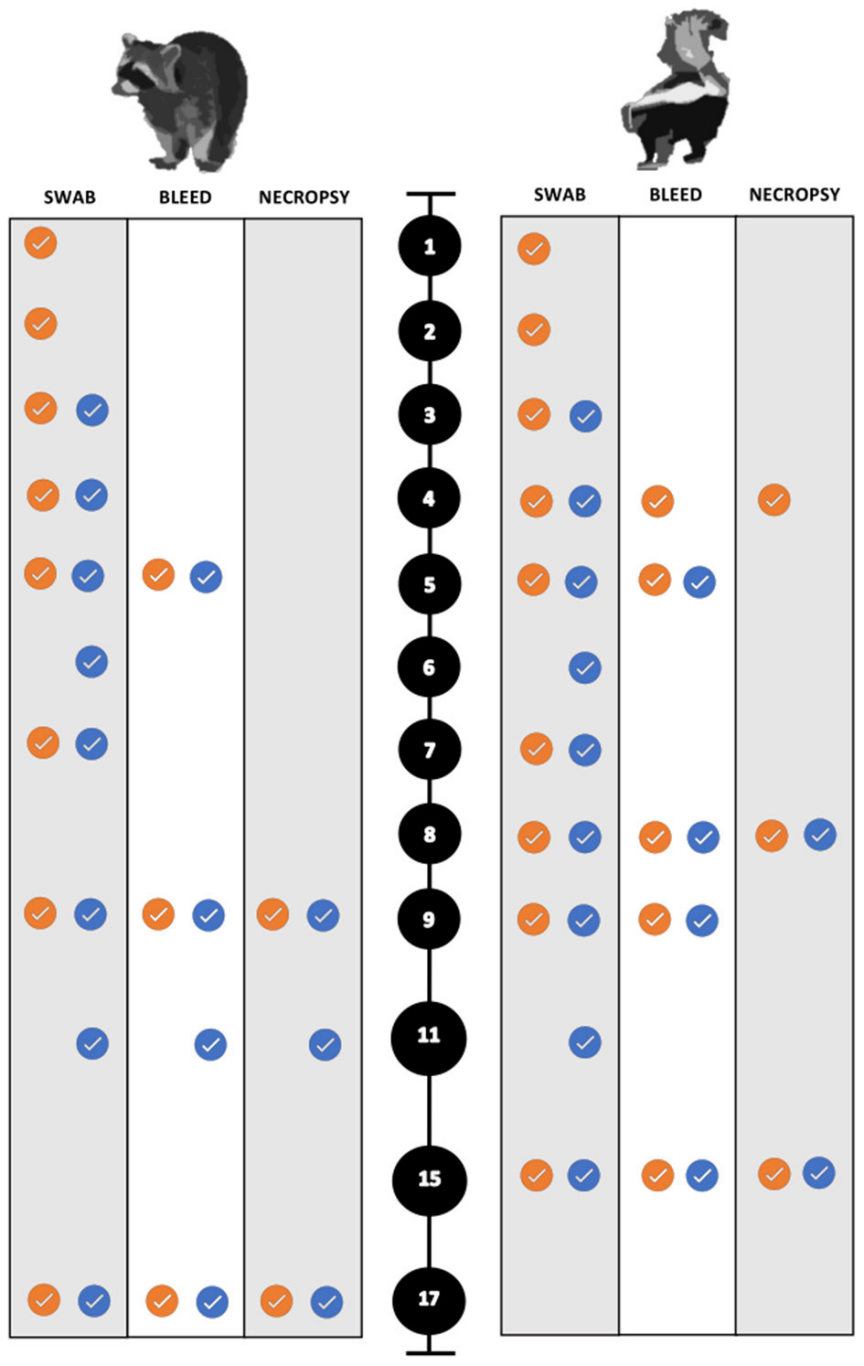
Sampling scheme and timing for the experimental SARS-CoV-2 infection trials of both raccoons and striped skunks. The black circles represent days post inoculation (dpi). The orange circles indicate directly inoculated (DI) animals. The blue circles indicate the direct contact (DC) animals.

For the collection of nasal and rectal swabs, animals were physically restrained and sedated with 30–45 mg/kg trazodone PO (Cadila Healthcare Ltd., India) suspended in either water or equal parts of ORA-Plus® Oral suspending vehicle and ORA-Sweet® (Perrigo, USA) in a syringe. The blood and swab collection scheme for both species is summarized in Figure 2. To evaluate environmental transmission, swabs of food and water bowls were obtained each sampling period prior to any animals being handled. Swabs and blood samples were also collected for all animals when euthanized.

Nasal swabs were obtained by swabbing both sides of the nasal passage using a single sterile polyester swab (Puritan, USA). Rectal swabs were obtained using sterile cotton swabs (Medline Industries, Inc., China). All swabs were placed in 1.5 mL cryovials (SealRite®) with 1 mL of Dulbecco's sterile phosphate-buffered saline (dPBS) (Sigma-Aldrich, Co.) for raccoons and 1 mL of sterile virus isolation medium composed of MEM for skunks. The blood samples and swabs were maintained in an insulated container with frozen gel packs until stored, and the whole blood was centrifuged within 1 h for the collection of serum. All swabs and serum were then stored at−80°C until processed.

Animals were anesthetized, euthanized, and necropsied at predetermined intervals to maximize the chance of detecting histopathologic changes during the course of infection. All animals were sampled as described above after humane euthanasia. Animals were anesthetized with dexmedetomidine (0.04 mg/kg) (Dexdomitor™), butorphanol (0.2 mg/kg) (Torbugesic™), and ketamine (5 mg/kg) (Zetamine™, OneVet, USA), and euthanized with an intracardiac dose of sodium pentobarbital (0.25 mL/kg) (Euthanasia Solution, Med-Pharmex Inc., USA). All animals were necropsied the day of euthanasia. Raccoons were euthanized on 9 dpi (*n* = 5; two DI from each dose group, and a control), on 11 dpi (*n* = 3; one DC one from each dose group, one control). The remaining experimental and control raccoons were euthanized and necropsied on 17 and 18 dpi, respectively. The experimental infection trials were performed first on raccoons. Due to the unremarkable nature of the raccoon gross necropsies, the necropsy interval was changed for skunk infection trials. Necropsies were performed earlier to capture early and subtle pathologic lesions such that skunks were euthanized on 4 dpi, (*n* = 3; two DI from each dose group, and a control), and on 8 dpi, (*n* = 4; two DI and two DC from each dose group) similar to Schlottau et al. and Freuling et al. (9, 52). A control skunk was euthanized on 7 dpi for comparison. The remaining control and experimental skunks were euthanized and necropsied on 14 and 15 dpi, respectively (Figure 2).

### Sample Analysis

#### Virus Isolation and Molecular Testing

Swab samples were placed in individual microcentrifuge tubes containing 1 mL of viral media, vortexed, and then centrifuged at 10,000 rpm for 10 min. Supernatant (100 μL) from each tube was inoculated into a separate well on a 12-well plate seeded with 3-to-4-day old Vero E6 cell culture monolayers. The plates were observed daily for cytopathic effect (CPE) for 10 days. If CPE was evident, the cell culture supernatant was collected and tested for the presence of SARS-CoV-2. Viral RNA (vRNA) was extracted from positive samples using the QIAamp Viral RNA Mini Kit (Qiagen Inc.), following the manufacturer's protocol. A validated real-time reverse transcription PCR (rRT-PCR) protocol was used for detection of SARS-CoV-2 (53, 54). Reactions were conducted on a Step OnePlus Real-Time PCR System (Applied Biosystems, Inc.).

The same rRT-PCR protocol was used as described above to evaluate tissues and nasal, fecal, and environmental swab samples for the presence of SARS-CoV-2 RNA. A positive rRT-PCR result was defined as the detection of both the N1 and N2 genes. Both the N1 and N2 primer/probe had to have a cycle threshold (Ct) of ≤ 35 to be considered positive for the presence of SARS-CoV-2 RNA. Samples evaluated that resulted in a Ct of >35 for both probes were considered negative and samples with a Ct of ≤ 35 for one probe and a Ct of >35 for the other probe were also considered negative as reported in Shriner et al. (41). Viral stock with a titer of 10^5^ pfu/200 ul was used as a positive control.

Skunk tissues (nasal conchae, tracheobronchial lymph node, tonsil, mid-length trachea, right middle lobe lung, heart, kidney, and jejunum) and select raccoon tissues (tracheobronchial lymph node, tonsil, right middle lobe lung) samples were homogenized with gentleMACS™ C Tubes (Miltenyi Biotec Inc., Germany) using a gentleMACS™ Dissociator (Miltenyi Biotec Inc.). Tubes were then centrifuged at 3,220 rpm for 10 min at 22°C. Then, 100 μL of supernatant from each tube was inoculated into a separate well on a 12-well plate seeded with 3-to-4-day old Vero E6 cell culture monolayers. CPE was determined as discussed above. An additional 140 μL of supernatant from each tube was collected and tested for the presence of SARS-CoV-2 using the extraction protocol and rRT-PCR protocol listed above.

#### Plaque Assays to Quantify Virus

Cell culture supernatant (200 μL) from samples that were positive for SARS-CoV-2 via VI and rRT-PCR was diluted 10-fold (with the first well containing no dilution) for a series of 5 dilutions (10^−1^, 10^−2^, 10^−3^, 10^−4^, 10^−5^), inoculated into a 6-well plate previously seeded with 4-day old Vero E6 cell culture monolayers, and incubated at 37°C and 5% CO_2_ for 1 h. Each well was then overlaid with 4 mL of a gum tragacanth overlay solution (equal parts 2% gum tragacanth & 2XMEM, supplemented with 2 mL of Fetal Bovine Serum (FBS), 5 mL of Antibiotic Antimycotic Solution) and allowed to incubate as described above for 7 to 10 days. Once plaques were noted grossly, each cell culture was inactivated with 10% formalin and crystal violet solution and allowed to fix for 24 to 48 h. Once cells were fixed, SARS-CoV-2 titers (log_10_ PFU/mL) were evaluated in wells for which more than one plaque was present; no plaques were seen past 10^−3^ dilution on any sample. As previously determined, a ½ log is lost for each freeze thaw cycle, and all vials had been through 2 cycles, presumptively decreasing titers by 1 log (D.G. Mead and E.R. Lafortaine, unpublished data).

### Microtitration Serum Neutralization

SARS-CoV-2 neutralizing antibodies were detected and quantified using serum microneutralization. Serum samples were heat-inactivated at 56°C for 30 min. Then, samples were 2-fold serially diluted in duplicates from 1:4 to 1:256 and incubated at 37°C and 5% CO_2_ with 100 TCID_50_ of the same strain of virus used in the inoculum in 96-well plates for 1 h. The wells were then overlaid with 150 μL of Vero E6 cells. The plates were incubated as described above and observed for CPE daily for 7 to 10 days, after which sample neutralization endpoint titers were determined.

### Necropsy, Histology, and Immunohistochemistry

All inoculated and control animals were necropsied within 2 h of euthanasia. Approximately 0.5 cm^3^ samples of nasal conchae, tracheobronchial lymph node, tonsil, mid-length trachea, right middle lobe lung, heart, kidney, and jejunum were placed in cryovials and stored at −80°C for subsequent laboratory analyses. Additional samples collected into 10% neutral buffered formalin for histopathologic evaluation included nasal sinus, trachea, left cranial and caudal lung lobes, right cranial and middle lung lobes, bronchus, lymph nodes (tracheobronchial, retropharyngeal, prescapular, and mesenteric), tonsil, tongue, esophagus, duodenum, jejunum, ileum, stomach, large intestine, left lateral liver lobe, gall bladder, pancreas, spleen, heart, kidney, thymus, thyroid gland, adrenal gland, gonad, skeletal muscle (biceps), urinary bladder, bone marrow, cerebrum, cerebellum, brainstem, and eye.

Once fixed, nasal sinus tissues were transferred to 12.5% neutral EDTA solution (250 g EDTA disodium salt (J.T. Baker Inc. USA), 1,750 mL distilled water, and 25 g sodium hydroxide) where they were allowed to decalcify for 14 to 21 days. Fixed tissues were routinely processed, embedded in paraffin wax, and 4 μm thick sections were stained with hematoxylin and eosin (HE). Duplicate slides with deep nasal sinus, mid-trachea, left cranial lung lobe, bronchus, tracheobronchial and prescapular lymph nodes, tonsil, and jejunum for all raccoons inoculated with low and high SARS-CoV-2 doses also underwent immunohistochemistry (IHC) for SARS-CoV-2 antigen. These same tissues, in addition to frontal nasal sinus, left caudal lung lobe, right cranial lung lobe, retropharyngeal lymph node, kidney and heart also underwent IHC for all skunks inoculated with low and high doses, as well as the two high dose direct contact skunks.

IHC was performed on an automated stainer (IntelliPATH, Biocare Medical, USA). A rabbit polyclonal antibody for SARS-CoV-2 (ThermoFisher, PA141098) at a dilution of 1:100 for 60 min was used. Antigen retrieval on tissue sections was achieved using Citrate Solution 10X (BioGenex, Fremont, USA) at a 1:10 dilution 10 for 15 min at 110°C. A biotinylated goat anti-rabbit antibody at a 1:100 dilution (Vector Laboratories, USA) was utilized to detect the target, and immunoreaction was visualized using Warp Red Chromogen (Biocare Medical) for 10 min and counterstained with hematoxylin. A cell pellet with infected cells was used as a positive control. All histology and immunohistochemistry were performed at the Athens Veterinary Diagnostic Laboratory at the University of Georgia and slides were read blindly by a board-certified veterinary pathologist.

## Results

None of the experimental animals of either species developed a fever (e.g., rectal temperature > 39.2°C), lost weight, changed behavior or displayed any signs of clinical disease throughout the study. Viral shedding was only detected on nasal swabs by virus isolation from two high dose DI skunks (H2R on 3, 4, and 5 dpi and H1L on 1 and 2 dpi) (Table 1). The highest amount shed was 3.3 log_10_/mL on 4 dpi. Virus was not isolated from any raccoon swabs, raccoon tissues, skunk rectal swabs, or skunk tissues.

**Table 1:** SARS-CoV-2 virus isolation data for two striped skunks (*Mephitis mephitis*) that shed virus after intranasal inoculation.

**ID**	**Contact**	**Days post inoculation (dpi)**				
Skunks^*^		**1**	**2**	**3**	**4**	**5**
H1-L	Directly inoculated	2	3.2			
H2-R	Directly inoculated			2.8	3.3	^**^0

*Values presented in plaque forming units (log_10_/mL)*.

* *Both H1-L and H2-R were inoculated with a high dose [10^5^ plaque forming units (PFU)] of SARS-CoV-2. No animals were found to shed virus after 5 dpi*.

** *The culture for H2-R developed cytopathic effect (CPE) on 5 dpi, however, no plaques were seen after staining*.

All skunk and select raccoon samples were evaluated for the presence of vRNA using rRT-PCR (Ct of ≤ 35). SARS-CoV-2 RNA was detected in the nasal swabs of 3/8 DI raccoons (H1R, H2L, L2L) and 4/8 DI skunks (H1L, H1R, H2R, L2R); this includes two skunks, H1-L and H2-R, from which virus was isolated as displayed in Table 2. In addition, skunks H1-L and H2-R were the only animals in this study to have Ct values ≤ 28, a threshold that coincides with historic human data for obtaining culturable virus when evaluating the samples for the SARS-CoV-2 N gene via RT-PCR (Supplementary Table 2) (55). Viral RNA was also detected from one high dose DI skunk (H2R) nasal turbinate tissue sample from 8 dpi. No vRNA was detected from skunk or raccoon rectal swabs, skunk environmental swabs, or raccoon tissues. All directly inoculated animals of both species seroconverted—defined by a 4-fold increase in antibody titers—by the end of the study; however, no seroconversion occurred in any direct contact animals. The highest titer was 1:64 in raccoons and 1:128 in skunks, and the earliest seroconversion timepoint was on 9 dpi and 8 dpi, respectively (Supplementary Table 1).

**Table 2:** SARS-CoV-2 RNA detection in nasal swabs of intranasally direct inoculated (DI) and direct contact (DC) raccoons (*Procyon lotor*) and striped skunks (*Mephitis mephitis*) by real-time reverse transcriptase PCR (rRT-PCR).

**DPI**	Raccoon^*^		Skunk^**^	
**Exposure**	**Direct**	**Contact**	**Direct**	**Contact**
1	3/8		2/8	
2	0/8		4/8	
3	0/8	0/4	3/8	0/4
4	0/8	0/4	2/8	0/4
5	0/8	0/4	2/6	0/4
6		0/4		0/4
7	0/8	0/4	0/7	0/4
8			0/2	0/2
9	0/8	0/4	0/4	0/2
11		0/4		0/2
15			0/4	0/2
17	0/4	0/2		
Total seroconversion	8/8	0/4	8/8	0/4

* *Of the rRT-PCR positive raccoons, 2/3 belonged to the DI high dose groups and 1/3 belonged to the DI low dose group*.

** *Of the rRT-PCR positive skunks, 3/4 belonged to the DI high dose group and 1/4 belonged to the DI low dose group*.

The gross necropsies for all animals were unremarkable. No microscopic lesions or SARS-CoV-2 specific immunohistochemical labeling were evident in tissues from raccoons. The frontal and deep nasal conchae of three DI high dose skunks (H1R, H2R, H2L) and two DI low dose skunks (L1R, L2L) had mildly to moderately increased numbers of widely scattered lymphocytes and plasma cells in the superficial lamina propria vs. DC and control animals. At least one of four examined sections of lung, either the left cranial/caudal or right cranial/middle lung lobes, of DI high dose skunks (H1R, H2R, H1L) and DI low dose skunks (L2R, L2L) had mildly increased numbers of perivascular lymphocytes and plasma cells randomly scattered throughout the interstitium. There was no corresponding immunohistochemical labeling in these nor in any other tissues examined from skunks. Incidentally, all skunks had moderate to severe, diffuse hepatic lipidosis and one DI skunk (L1R) had focal, purulent rhinitis in the frontal nasal conchae. All skunks had robust lymphoid tissue in lymph nodes, spleen, bronchus-associated lymphoid tissue (BALT; lungs), and gastrointestinal-associated lymphoid tissue (GALT; intestine).

## Discussion

A One Health approach to understand and mitigate the epidemiology and management of SARS-CoV-2 in a wide range of hosts calls for a collaborative effort between governmental agencies, academic and private institutions, and the public (56). Such efforts must include experimental trials, wildlife health assessments, and pathogen surveillance (57). In terms of the number of human infections and deaths and the currently known animal host range, the COVID-19 pandemic is one of the most important global emerging zoonoses to date, which will take a concerted multidisciplinary approach to manage.

Our findings demonstrate that while striped skunks and raccoons are susceptible to SARS-CoV-2 infection, it is unlikely that either species is likely to be a competent reservoir for SARS-CoV-2 in a natural setting. No animals experienced clinical disease during this study. Raccoons exhibited no pathology, and skunks had mild evidence of subclinical recovering cellular response to viral infection in the nasal conchae and lungs. The lack of virus isolation from raccoons, evidence of direct transmission between both species, and low amount of virus shed by skunks would likely impede the virus's ability to establish in wild populations. Similar results of an experimental trial with skunks were reported by Bosco-Lauth et al. (7). Of 6 striped skunks that were intranasally inoculated with approximately 10^5^ PFU of SARS-CoV-2, three shed virus up to 7 dpi, the highest amount of which was 2.3 log_10_ pfu/swab. They also isolated virus from the nasal turbinates of 2/3 skunks euthanized on 3 dpi, similar to the vRNA detected in a nasal turbinate tissue sample of a skunk euthanized on 8 dpi in our study. Also similar to our study, all inoculated skunks seroconverted. Bosco-Lauth et al. (7) also inoculated three raccoons, none of which were positive by VI or RT-PCR. In our study, all of our inoculated raccoons seroconverted whereas none of the raccoons in Bosco-Lauth et al. (7) seroconverted. The reason for this difference is unknown but could be due to the use of different serological assays. Another important difference between these two studies is that we included naïve contact animals to test cage-mate transmission, which provided data on the potential for SARS-CoV-2 to circulate in striped skunk and raccoon populations.

We describe viral RNA using presence/absence data similar to methods for SARS-CoV-2 animal reporting by the USDA (41, 58, 59). We also provide data on the detection of infectious virus (by plaque assay) to assist in assessing the potential for transmission of SARS-CoV-2 in nature. In some cases, virus quantification by PCR is used to estimate or determine virus quantities; however, this could lead to confusion as to how results translate to natural infections among wild and captive animals (55, 60, 61). In our study, virus was isolated from samples from two skunks (H1-L and H2-R) between 1 and 5 dpi; the corresponding Ct decreased notably between 3 and 4 dpi before steadily increasing (Supplementary Table 2). This trend may reflect diminished viral replication as Ct values increased. While rRT-PCR is a sensitive diagnostic tool to evaluate for the presence of SARS-CoV-2, it should be used in conjunction with additional assays such as virus isolation to strengthen inferences about transmission potential and other epidemiological factors in wildlife and others.

As with many susceptibility trials with wildlife, especially those that require high containment housing, availability, logistical challenges, and animal welfare considerations often limit sample size (62, 63). Given our studies small sample size and our animal sourcing, our findings may not readily translate into the susceptibility of wild populations due to factors such as senescence, immunocompetence (i.e., parasite burden, environmental conditions, gestation) and co-infections (e.g., canine distemper virus, parvovirus, etc.). Also, the rapid emergence of increasingly infectious SARS-CoV-2 variants in human populations presents new possibilities, such as increased transmissibility to previously marginally susceptible or unsusceptible species (64). Emerging variants of concern, B.1.1.7 and B.1.617.2, have been isolated from both companion animals (e.g., domestic dogs and cats) and captive wildlife (e.g., lions in a zoological institution), respectively (65–67). However, to date, no variants of concern have been isolated from free ranging wildlife, nor used in experimental infection trials of wildlife species, thus it is difficult to infer what impact these emerging variants will have on free-ranging raccoons and striped skunks. Despite evidence of poor transmission, care should be taken to avoid transmission of SARS-CoV-2 to skunks and raccoons in a captive setting (e.g., zoological institutions, rehabilitation centers) where close encounter with individuals shedding different strains and high viral loads may influence outcomes. In this study, all raccoons and skunks seroconverted after direct inoculation with SARS-CoV-2, however only a small subset of these animals shed detectable viral RNA (*n* = 7), and even less shed viable virus (*n* = 2). Moreover, these results suggest that seroprevalence studies may be the most sensitive large-scale approach for determining COVID-19 exposure in susceptible wildlife contrary to current PCR-based animal surveillance in the US (59). However, the lack of viral shedding in raccoons or select skunks highlight that future wildlife surveillance studies should interpret antibody presence with caution, as seroconversion is not indicative of an animal having a profound role in the epidemiology of SARS-CoV-2.

While it seems unlikely for SARS-CoV-2 to circulate in raccoon and skunk populations, other taxonomically related species, such as several species of mustelids including various otters, weasels, badgers, and martens; especially species of particular conservation concern, like black-footed ferrets (*Mustela nigripes*), European mink (*Mustela lutreola*), giant otters (*Pteronura brasiliensis*), and sea otters (*Enhydra lutris*) have yet to be studied. Infection of highly susceptible species held in captive breeding programs, could result in outbreaks, hampering reintroduction efforts. For example, these concerns, in part, led to the majority of the captive breeding population of black-footed ferrets at the National Black-Footed Ferret Conservation Center outside Fort Collins, Colorado to be immunized with an experimental vaccine early in the COVID-19 pandemic (68). Continued global outbreaks of SARS-CoV-2 in farmed mink (69), sporadic reports of infection in domestic animals (36, 70–72), and detected spillover into captive and free-living wildlife populations [e.g., wild and escaped mink in Utah; (41, 73), various species including tigers, gorilla, and otters in zoological collections; (44, 74, 75)] highlight that additional research including further exploration of the drivers, ecological pathways, and susceptibility of SARS-CoV-2 in wildlife, especially Musteloidea, are needed.

## Data Availability Statement

The original contributions presented in the study are included in the article/Supplementary Material, further inquiries can be directed to the corresponding authors.

## Ethics Statement

The animal study was reviewed and approved by the University of Georgia's IACUC Committee (A2020 04-016) and Office of Biosafety (2020 0048).

## Author Contributions

SH, DM, NN, RF, and MY contributed to study design and implementation. SH, DM, SB, NN, MY, and RF participated in the experimental trials and sampling. DM, KA, SB, and RF processed samples. NN, DM, KA, and RF interpreted the data. RF wrote the first draft of the manuscript. SH, MY, and NN both contributed large portions to the manuscript. All authors contributed to manuscript revision, read, and approved the submitted version.

## Funding

Primary funding was provided by a National Science Foundation RAPID award (#2032044); graduate student assistantship support was additionally provided by an NSF EEID grant (#1518611). Additional support was provided by the member states wildlife management agencies of the Southeastern Cooperative Wildlife Disease Study through the Federal Aid to Wildlife Restoration Act (50 Stat. 917) and the Ecosystems Mission Area, United States Geological Survey, United States Department of Interior.

## Conflict of Interest

The authors declare that the research was conducted in the absence of any commercial or financial relationships that could be construed as a potential conflict of interest.

## Publisher's Note

All claims expressed in this article are solely those of the authors and do not necessarily represent those of their affiliated organizations, or those of the publisher, the editors and the reviewers. Any product that may be evaluated in this article, or claim that may be made by its manufacturer, is not guaranteed or endorsed by the publisher.
