# Supplementary material for: Experimental Susceptibility of North American Raccoons (Procyon lotor) and Striped Skunks (Mephitis mephitis) to SARS-CoV-2
Source: Front Vet Sci. 2022 Jan 12;8:715307. doi: 10.3389/fvets.2021.715307 (PMC8790025; doi:10.3389/fvets.2021.715307)
Supplement: Supplementary file 1 [file Data_Sheet_1.PDF]

Supplementary Material

1 Supplementary Figures and Tables

Supplementary Table 1. Serum neutralizing antibody development in striped skunks and raccoons intranasally inoculated with SARS-CoV-2, direct contact striped skunks and raccoons, and control striped skunks and raccoons.

ID	Contact	Detection*	DPI								
Skunks		Method	Baseline	4	5	7	8	9	10	11	Study End
C1-L	Control		1:4–1:8		1:4	**1:4**					
C1-R	Control		1:4					1:4–1:8			**1:4–1:8**
C2-L	Control		1:4–1:8					1:4			**1:4–1:8**
C2-R	Control		1:8	**1:4–1:8**							
L1-L	L-DI		1:4		1:4–1:8		**1:8–1:16**				
L1-R	L-DI		1:4–1:8	**1:4–1:8**							
L1-N	DC		1:4		1:4–1:8		1:4–1:8	**1:4–1:8**			
L2-L	L-DI		1:4		1:4–1:8			1:16–1:32			**1:32**
L2-R	L-DI	+; rRT-PCR	1:4					1:32–1:64			**1:64**
L2-N	DC		1:4					1:4–1:8			**1:4-1:8**
H1-L	H-DI	+; VI, rRT-PCR	1:4–1:8					1:32–1:64			**1:128**
H1-R	H-DI	+; rRT-PCR	1:4					1:32–1:64			**1:64-1:128**
H1-C	DC		1:16-1:32					1:32			**1:16-1:32**
H2-L	H-DI		1:4	**NS**							
H2-R	H-DI	+; VI, rRT-PCR	1:4–1:8		1:8		**1:16–1:32**				
H2-C	DC		1:4		1:4–1:8		**1:4–1:8**				

Raccoons		Method	Baseline	4	5	7	8	9	10	11	Study End
C1-L	Control		1:4						1:8		**1:4-1:8**
C1-R	Control		1:4						1:4-1:8		**1:4**
C2-L	Control		1:4						1:4-1:8	**1:4**	
C2-R	Control		1:4–1:8					**1:4**			
L1-L	L-DI		1:8					1:16			**1:32**
L1-R	L-DI		1:4					**1:8-1:16**			
L1-N	DC		1:4					1:4-1:8			**1:4-1:8**
L2-L	L-DI	+; rRT-PCR	1:4					1:8-1:16			**1:16**
L2-R	L-DI		1:8					**1:16**			
L2-N	DC		1:4–1:8					1:4		**1:4-1:8**	
H1-L	H-DI		1:4					1:8-1:16			**1:16-1:32**
H1-R	H-DI	+; rRT-PCR	1:4					**1:8-1:16**			
H1-C	DC		1:8-1:16					1:8			**1:8-1:16**
H2-L	H-DI	+; rRT-PCR	1:8					1:16-1:32			**1:64**
H2-R	H-DI		1:4					**1:16**			
H2-C	DC		1:8					1:4-1:8		**1:4-1:8**	

Table Footnotes: Bolded titers represent the terminal sample collected from an animal. End of study samples for remaining DI and DC raccoons were collected on DPI 17 and remaining control raccoons on DPI 18. End of study samples for remaining DI and DC skunks were collected on DPI 15 and remaining control skunks on DPI 14. *The “+” in the detection column indicates if SARS-CoV-2 was isolated in any animal via rRT-PCR or VI at any point of the study.

NS, No viable terminal sample was recovered due to hemolysis; DPI, day post inoculation; L-DI, Low Dose Directly Inoculated; H-DI, High Dose Directly Inoculated; DC, Direct Contact; VI, virus isolation; rRT-PCR, real-time reverse transcription PCR.

Supplementary Table 2. SARS-CoV-2 presence/absence Real-Time Reverse Transcriptase PCR results in striped skunks and raccoons intranasally inoculated with SARS-CoV-2 and direct contact striped skunks and raccoons.

ID	Contact	Swab	N1 Probe Ct Values									
**Skunks**			**1**	**2**	**3**	**4**	**5**	**6**	**7**	**9**	**11**	**15**
L1-L	L-DI	Nasal	NEG	NEG	NEG	NEG	NEG		NEG			
		Rectal	NEG	NEG	NEG	NEG			NEG			
L1-R	L-DI	Nasal	NEG	38.48		40.95						
		Rectal		37.84		NEG						
L1-N	DC	Nasal			39.44	NEG	NEG	NEG	38.62			
		Rectal			NEG	NEG	NEG	38.77				
L2-L	L-DI	Nasal	39.68	NEG	NEG	38.44	38.35		39.38	NEG		37.35
		Rectal	NEG	39.78	38.52	37.97	36.41		NEG	NEG		NEG
L2-R	L-DI	Nasal	38.46	31.41	NEG	NEG	33.95		37.95	NEG		37.92
		Rectal	38.93	NEG	39.66	NEG	NEG		NEG	NEG		NEG
L2-N	DC	Nasal			NEG	NEG	NEG	38.41		39.04	35.7	38.95
		Rectal			NEG	NEG	NEG	38.62	38.64	NEG	NEG	NEG
H1-L	H-DI	Nasal	35.29	31	29.27	33.87	29.87		36.22	NEG		NEG
		Rectal	35.46	NEG	NEG	39.74	NEG		NEG	35.92		38.63
H1-R	H-DI	Nasal	35.4	35.92	34.89	NEG	NEG		NEG	35.98		NEG
		Rectal	NEG	38.95	NEG	37.95	38.69		NEG	NEG		NEG
H1-C	DC	Nasal			37.21	NEG	NEG	39.47	NEG	NEG	39.04	37.55
		Rectal			35.39	NEG	38.63	NEG	NEG	NEG	39.56	NEG
H2-L	H-DI	Nasal	38.07	36.92	37.82	39.5						
		Rectal	NEG	36.82	37.63	NEG						
H2-R	H-DI	Nasal	35.39	34.19	29.86	26.81	30.55		37.36			
		Rectal	NEG	NEG	NEG	38.6	38.69		NEG			

H2-C	DC	Nasal			38.57	NEG	NEG	37.32	NEG			
		Rectal			39.64	NEG	NEG	NEG	NEG			

ID	Contact	Swab	N2 Probe Ct Values									
**Skunks**			**1**	**2**	**3**	**4**	**5**	**6**	**7**	**9**	**11**	**15**
L1-L	L-DI	Nasal	NEG	NEG	NEG	41.1	NEG		NEG			
		Rectal	NEG	NEG	NEG	NEG			NEG			
L1-R	L-DI	Nasal	39.47	NEG		NEG						
		Rectal		NEG		NEG						
L1-N	DC	Nasal			40.13	42.04	NEG	NEG	39.25			
		Rectal			40.71	NEG	40.68	42.01				
L2-L	L-DI	Nasal	42.02	NEG	NEG	39.99	36.8		NEG	40.56		41.01
		Rectal	40.26	NEG	NEG	NEG	39.05		NEG	NEG		NEG
L2-R	L-DI	Nasal	NEG	31.51	NEG	NEG	38.06		NEG	40.3		40.91
		Rectal	NEG	NEG	NEG	NEG	NEG		NEG	NEG		NEG
L2-N	DC	Nasal			40.83	NEG		NEG		39.14	39.7	44.86
		Rectal			NEG	NEG	NEG	NEG	NEG	NEG	NEG	NEG
H1-L	H-DI	Nasal	35.15	30.5	28.68	33.89	31.4		36.97	41.99		41.61
		Rectal	37.76	NEG	42	40.68	NEG		NEG	41.33		40.91
H1-R	H-DI	Nasal	34.76	35.37	33.95	NEG			NEG	40.48		NEG
		Rectal	NEG	NEG	39.92	NEG	NEG		NEG	NEG		NEG
H1-C	DC	Nasal			39.74	38.91		NEG	39.19	NEG	39.97	NEG
		Rectal			37.13	NEG	39.89	NEG	NEG	NEG	NEG	NEG
H2-L	H-DI	Nasal	35.95	35.48	NEG	41.16						
		Rectal	NEG	39.58	NEG	40.26						
H2-R	H-DI	Nasal	38.16	33.78	32.01	27.12	32.32		38.82			
		Rectal	NEG	NEG	41.4	NEG	NEG		NEG			

H2-C	DC	Nasal	NEG	NEG	NEG	40.29	NEG
		Rectal	NEG	NEG	NEG	NEG	41.13

ID	Contact	Swab	N1 Probe Ct Values									
**Raccoons**			**1**	**2**	**3**	**4**	**5**	**6**	**7**	**9**	**11**	**17**
L1-L	L-DI	Nasal	35.41	35.69	36.16	36.29	37.86		36.19	37.95		36.31
		Rectal	34.32	35.78	NEG	NEG	37		35.99	36.79		NEG
L1-R	L-DI	Nasal	35.14	35.89	37.05	36.02	35.82		37.24	36.29		
		Rectal	NEG	NEG	NEG	38.99	NEG		NEG	36.19		
L1-N	DC	Nasal			38.65	35.96	35.59	37.22	36.37	35.45	37.84	35.36
		Rectal			37.13	NEG	38.02	NEG	39.04	NEG	38.71	38.81
L2-L	L-DI	Nasal	33.64	35.38	37.43	36.4	35.85		NEG	37.97		36.49
		Rectal	34.37	NEG	38.13	36.53	37.04		37.2	NEG		37.97
L2-R	L-DI	Nasal	35.2	35.01	NEG	38.62	NEG		34.14	37.2		
		Rectal	40.99	NEG	NEG	36.98	NEG		37.14	38.79		
L2-N	DC	Nasal			NEG	35.03	36.6	36.43	36.09	37.84	35.14	
		Rectal			NEG	37.04	36.32	35.75	36.16	NEG	37.64	
H1-L	H-DI	Nasal	37.56	36.98	34.31	35.9	35.28		NEG	36.2		35.31
		Rectal	34.13	34.7	NEG	NEG	36.95		35.88	34.42		37.25
H1-R	H-DI	Nasal	35.12	38.78	35.46	36.89	38.9		37.46	37.28		
		Rectal	34.71	NEG	35.47	37.15	37		38.91	NEG		
H1-C	DC	Nasal			36.37	35.87	38.47	36.96	38.96	38.76	38.07	36.41
		Rectal			NEG	NEG	NEG	38.13	38.25	NEG	35.77	36.16
H2-L	H-DI	Nasal	34.96	36.13	36.63	34.44	38.41		36	37.27		34.77
		Rectal	34.39	NEG	34.75	NEG	NEG		37.69	38.77		39.07
H2-R	H-DI	Nasal	34.82	36.46	NEG	35.64	37.29		34.85	NEG		
		Rectal	35.34	35.32	35.33	NEG	NEG		37.29	37.45		
H2-C	DC	Nasal			34.92	35.35	37.96	35.2	38.95	36.41	35.41	

		Rectal			35.47	38.1	NEG	36.81	36.81	38.77	38.98	
**ID**	**Contact**	**Swab**	**N2 Probe Ct Values**									
**Raccoons**			**1**	**2**	**3**	**4**	**5**	**6**	**7**	**9**	**11**	**17**
L1-L	L-DI	Nasal	37.36	39.31	40.28	38.54	40.81		38.51	39.54		NEG
		Rectal	NEG	41.1	NEG	38.14	NEG		NEG	40.24		NEG
L1-R	L-DI	Nasal	40.08	39.18	42.06	37.39	38.06		41.08	36.95		
		Rectal	41.5	NEG	40.62	39.8	41.68		39.21	37.46		
L1-N	DC	Nasal			40.3	38.87	36.35	38.76	38.19	40.28	38.11	39.65
		Rectal			NEG	NEG	NEG	41.99	40.97	42.78	NEG	NEG
L2-L	L-DI	Nasal	35.73	37.85	38.93	38.55	36.42		39.75	39.16		NEG
		Rectal	39.38	NEG	NEG	42.73	39.09		NEG	NEG		40.05
L2-R	L-DI	Nasal	37.58	37.86	40.88	42.03	36.76		36.78	40.63		
		Rectal	NEG	40.69	NEG	40.26	NEG		42.28	40.14		
L2-N	DC	Nasal			37.87	36.18	37.86	38.23	37.65	NEG	41.31	
		Rectal			NEG	NEG	NEG	NEG	NEG	42.04	NEG	
H1-L	H-DI	Nasal	36.51	40.47	37.48	36.62	36.55		38.95	38.57		NEG
		Rectal	41.99	NEG	38.22	NEG	38.53		NEG	37.4		NEG
H1-R	H-DI	Nasal	35.61	40.67	41.08	37.07	39.47		38.99	36.92		
		Rectal	NEG	42.79	NEG	NEG	42.05		37.3	38.99		
H1-C	DC	Nasal			NEG	41.4	40	37.08	41.66	39.73	NEG	39.13
		Rectal			NEG	40.33	NEG	NEG	39.11	NEG	37.26	40.12
H2-L	H-DI	Nasal	35.48	38.53	40.53	36.44	37.1		37.76	NEG		NEG
		Rectal	40.01	40.25	39.24	38.11	40.68		40.2	43.7		42.02
H2-R	H-DI	Nasal	38.66	38.75	41.87	37.39	37.58		37.85	NEG		
		Rectal	NEG	38.63	40.82	40.67	NEG		NEG	NEG		
H2-C	DC	Nasal			36.6	36.63	39.2	36.6	40.93	NEG	39.88	

Rectal	39.28	NEG	36.98	38.22	38.22	NEG	41.07

Table Footnotes: The N1 and N2 primer/probe had to have a cycle threshold (Ct) of ≤35 to be considered positive (**GREEN**) for the presence of SARS-CoV-2 RNA. Samples evaluated that resulted in a Ct of >35 for both probes were considered negative (**RED**) and samples with a Ct of ≤35 for one probe and a Ct of >35 for the other probe (**YELLOW**) are also considered negative.

NEG, Negative; DPI, day post inoculation; L-DI, Low Dose Directly Inoculated; H-DI, High Dose Directly Inoculated; DC, Direct Contact; rRT-PCR, real-time reverse transcription PCR.

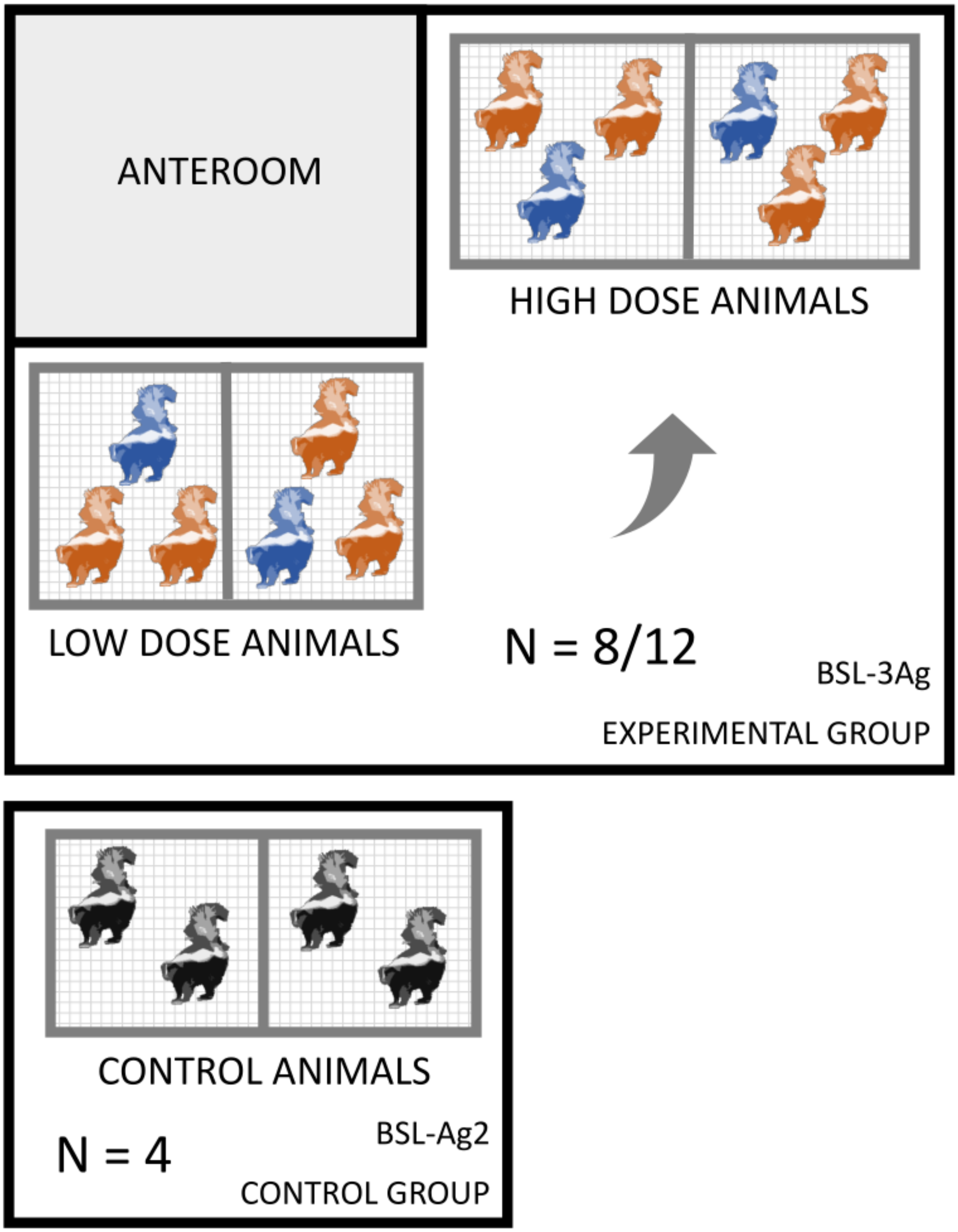

Supplementary Figure 1. Agriculture Biosafety Level 3 (BSL-3Ag) room layout for both raccoon and skunk infection trials. The orange skunks represent the directly inoculated animals and the blue skunks represent the direct contact animals. The room's unidirectional airflow is represented by the arrow and did not recirculate.
